# The Clip-Segment of the von Willebrand Domain 1 of the BMP Modulator Protein Crossveinless 2 Is Preformed

**DOI:** 10.3390/molecules181011658

**Published:** 2013-09-25

**Authors:** Juliane E. Fiebig, Stella E. Weidauer, Li-Yan Qiu, Markus Bauer, Peter Schmieder, Monika Beerbaum, Jin-Li Zhang, Hartmut Oschkinat, Walter Sebald, Thomas D. Mueller

**Affiliations:** 1 Julius-von-Sachs Institut für Biowissenschaften der Universität Würzburg, Julius-von-Sachs Platz 2, Würzburg D-97082, Germany; E-Mails: juliane.fiebig@botanik.uni-wuerzburg.de (J.E.F.); stella.keiper@biozentrum.uni-wuerzburg.de (S.E.W.); ma.bauer@botanik.uni-wuerzburg.de (M.B.); 2 Lehrstuhl für Physiologische Chemie II, Biozentrum der Universität Würzburg, Am Hubland, Würzburg D-97074, Germany; E-Mails: liyanqiu@biozentrum.uni-wuerzburg.de (L.-Y.Q.); jinli.zhang@mso.umt.edu (J.-L.Z.); sebald@biozentrum.uni-wuerzburg.de (W.S.); 3 Leibnizinstitut für Molekulare Pharmakologie (FMP), Campus Berlin-Buch, Robert-Roessle Str. 10, Berlin D-13125, Germany; E-Mails: schmieder@fmp-berlin.de (P.S.); beerbaum@fmp-berlin.de (M.B.); oschkinat@fmp-berlin.de (H.O.)

**Keywords:** bone morphogenetic proteins, TGF-β superfamily, BMP antagonist, protein-protein recognition, NMR spectroscopy, von Willebrand type C domain

## Abstract

Bone Morphogenetic Proteins (BMPs) are secreted protein hormones that act as morphogens and exert essential roles during embryonic development of tissues and organs. Signaling by BMPs occurs via hetero-oligomerization of two types of serine/threonine kinase transmembrane receptors. Due to the small number of available receptors for a large number of BMP ligands ligand-receptor promiscuity presents an evident problem requiring additional regulatory mechanisms for ligand-specific signaling. Such additional regulation is achieved through a plethora of extracellular antagonists, among them members of the Chordin superfamily, that modulate BMP signaling activity by binding. The key-element in Chordin-related antagonists for interacting with BMPs is the von Willebrand type C (VWC) module, which is a small domain of about 50 to 60 residues occurring in many different proteins. Although a structure of the VWC domain of the Chordin-member Crossveinless 2 (CV2) bound to BMP-2 has been determined by X-ray crystallography, the molecular mechanism by which the VWC domain binds BMPs has remained unclear. Here we present the NMR structure of the *Danio rerio* CV2 VWC1 domain in its unbound state showing that the key features for high affinity binding to BMP-2 is a pre-oriented peptide loop.

## 1. Introduction

Bone morphogenetic proteins (BMPs) are secreted protein hormones, which form a subgroup within the large Transforming Growth Factor-β (TGF-β) superfamily (for recent reviews see [1,2]). These factors exert essential functions during embryonic development, controlling early events such as gastrulation contributing to embryonic patterning processes [3,4] and are involved in the development of various organs and tissues [5,6,7,8,9,10]. Later in the adult organism BMPs regulate homeostasis of tissues and organs and are thus considered important target molecules for tissue engineering and regenerative medicine applications [8,11]. As observed in gene deletion studies in mice [12], loss of BMP function frequently leads to pathological conditions, such as skeletal malformation diseases [10,13], osteoporosis/osteopetrosis [14], cardiovascular and metabolic diseases, muscular disorders and cancer.

Signaling by BMPs is initiated by binding to two types of serine/threonine kinase transmembrane receptors termed type I and type II (for review [15]). Upon BMP-induced receptor oligomerization the type II receptor kinase phosphorylates the type I receptor kinase in a characteristic glycine-serine rich sequence motif (GS-box) thereby activating the type I receptor kinase. One downstream signaling cascade (among others) includes the so-called SMAD factors [a combined name from “small body size” (Sma) and “mothers against decapentaplegic” (MAD)]. Upon phosphorylation, receptor-associated SMADs (R-SMADs 1, 2, 3, 5 or 8) form heterotrimeric complexes with the common mediator SMAD (Co-SMAD 4) and translocate into the nucleus, where they regulate gene transcription of BMP-dependent genes. Signaling crosstalk—SMAD factors act as co-transcriptional regulators—with other signaling pathways such as Wnt/β-catenin, the LIF or the TNFa pathway likely ensure cell-specific functions of BMPs. Furthermore, additional, SMAD independent signaling cascades induced by BMPs exist namely the p38 MAP-kinase pathway and the PI3 kinase/Akt pathway (for a review [16]).

Due to their essential *in vivo* functions and due to the fact that BMPs function as morphogens—*i.e.*, the local growth factor concentration is quantitatively translated into a specific response—the signaling has to be tightly regulated. Thus it might come as a surprise that the more than 30 TGF-β protein hormones (of which 19 belong to the BMP subgroup) face only 12 receptors, seven type I and five type II receptors [1,2]. The consequence of this numeral discrepancy, a pronounced ligand-receptor promiscuity, *i.e.*, one particular receptor assembly is bound and activated by various ligand members, contrasts the expectation that each BMP hormone is able to transduce a highly specific (possibly even concentration-dependent) signal via a specific receptor [2]. Since *in vivo* situations exist, e.g., limb development (for a review see [17]), where different TGF-β factors with overlapping receptor-binding properties/receptor preference are present in the same cellular environment and at the same time, additional mechanisms must exist to allow transduction of ligand-specific signals. It thus is not surprising that signaling of TGF-β superfamily members is tightly regulated by a plethora of additional extra- and intracellular factors [18]. Intracellularly, inhibitory SMAD factors, ubiquitin ligases targeting BMP receptors and R-SMADs or anti-proliferative proteins like Tob or the transcriptional co-repressor Ski can downregulate signaling. However, the unprecedented number of extracellular TGF-β ligand-binding proteins capable to antagonize BMP activity is certainly a unique feature among the various cytokine/growth factor superfamilies. The diverse group of BMP modulator proteins comprises Noggin, Follistatin and Follistatin-related proteins, Twisted Gastrulation, the members of the Chordin family to which also Crossveinless 2 belongs, as well as the various members of the DAN and CCN families [18]. Although structure data is limited [19,20,21,22,23], the known architectures vary to a large degree showing barely any homology among the different families. However, the mechanism by which these modulator proteins antagonize ligand activity seems highly conserved. The structure of BMP antagonist Noggin bound to BMP-7 revealed this principle for the first time, showing that the dimeric Noggin blocks simultaneously the type I and type II receptor binding epitopes of the BMP ligand leading to efficient inhibition of BMP signaling [20]. Due to its monomeric nature, two molecules of the structurally vastly different Follistatin wrap around the homodimeric TGF-β ligand thereby similarly covering the receptor epitopes of Activin A [21,22].

To block both receptor epitopes in TGF-β ligands the modulator proteins do not need to be large as Noggin or Follistatin. Analyses of the Chordin modulator family have shown that a single small von Willebrand type C domain (VWC) comprising only 60 to 65 residues present in Crossveinless 2 (CV2, also known as BMP binding endothelial regulator, BMPER), Chordin-like 2 or Chordin, is sufficient to bind BMP-2 with high nanomolar affinities and to efficiently compete for binding to BMP type I and type II receptors [24]. The von Willebrand type C domain motif has been originally identified from the gene structure of the von Willebrand factor, a large multimeric plasma protein exerting an essential role in hemostasis [25]. With the development of new bioinformatics tools [26,27,28], the VWC domain could also be detected in various other extracellular proteins (e.g., the SMART database lists 87 human proteins [29], PFAM has 109 human sequence entries [30]) that serve highly diverse functions. Usually the VWC domain occurs in multi-domain proteins, often in multiple copies and together with various other protein domains. Further analysis has shown that the VWC is the key element in the Chordin family, the Nel and the CTGF/CCN families of BMP modulator proteins [31]. However, the low sequence similarity—only the 10 cysteine residues and their spacing are strictly conserved—have so far prevented deciphering the mechanism by which VWC domains recognize and bind BMPs. Conformingly, a study analyzing the BMP binding of members of the Chordin family containing multiple VWC domains showed that the binding affinities for BMP highly varies between these different VWC repeats with some VWC domains not even binding to BMPs at all [24]. Structure analysis of the N-terminal VWC domain of CV2 bound to BMP-2 then revealed for the first time how VWC domain might interact with BMP ligands [23]. Despite the small size of only 66 residues, the VWC domain of CV2 exhibits a modular tripartite architecture, with an N-terminal Clip segment and two between 25 and 30 residues sized subdomains termed SD1 and SD2. The binding of this small modular VWC protein domain to the BMP-2 ligand somewhat resembles a paperclip (CV2 VWC1) attached to a sheet of paper (BMP-2) [23]. The subdomain SD1 adheres to the β-strands of finger 2, which is part of the type II receptor epitope of BMP-2 and thus blocks binding to type II receptors. The N-terminal Clip segment folds into the so-called “wrist” epitope and blocks interaction with type I receptors. The BMP-interacting part of N-terminal Clip segments of CV2 VWC1 as well as of Noggin each comprise about six residues and act mechanistically highly similar by binding of the peptide stretch to a hydrophobic knob-into-hole motif, which presents one hot spot of binding for the BMP ligand-type I receptor interaction [20,23]. In spite of the similar interaction none of the residues in this Clip peptide stretch are conserved between CV2 VWC1 and Noggin. Furthermore, since the Clip segment of both modulator proteins/domains does not exhibit any secondary structure it has led to debate whether this Clip segment adopts a defined structure in its free unbound state or whether this element is flexible and disordered before forming a complex with the BMP ligand. However, mutagenesis studies on CV2 VWC1 showed that deletion of the N-terminal Clip segment results in an almost complete loss of BMP binding indicating that the interaction between the seemingly flexible Clip segment and BMP-2 strongly contributes to the overall tight binding [23]. Similarly, a mutation of proline 35 in Noggin, which is the residue in the Clip segment mimicking the hydrophobic knob-into-hole interaction of BMP-type I receptor interaction, to serine or arginine has been found in patients suffering from skeletal malformation disorders suggesting a loss of Noggin function likely due to loss of BMP binding [32,33,34]. This raises the question how the Clip segment can become a major contributor to the overall binding energy for interacting with BMP ligands, if we assume that due to amino acid sequence and lack of interaction with of this region with the structured rest of Noggin or CV2 VWC1 domain this short peptide stretch adopts its proper conformation just upon binding to the BMP ligand. As we expect that most of the binding energy released by interaction of this stretch with BMP is consumed by the loss of entropy required for fixation of this flexible stretch, either a highly cooperative binding mode exists with numerous non-covalent interactions between the Clip segment and the BMP ligand or the conformation of the Clip segment is unexpectedly at least partially preformed. To test the latter hypothesis we have determined the structure of the VWC1 domain of Crossveinless 2 of *Danio rerio* by NMR spectroscopy in its free unbound state.

## 2. Results and Discussion

### 2.1. Production of the N-Terminal von Willebrand Type C Domain of Crossveinless 2 for NMR Analysis

The BMP modulator protein of the Chordin family, Crossveinless 2 (CV2) is a modular multidomain protein (see also Figure 5a) consisting of five consecutive von Willebrand type C domains (numbers VWC1 to VWC5), each of them between 50 to 60 residues long, and with the first VWC domain directly starting after the signal peptide (*D. rerio* CV2 VWC1: 9–64; VWC2: 67–122; VWC3: 125–183; VWC4: 197–246; VWC5: 258–314). The VWC stretch is followed by a single von Willebrand type D (VWD) domain comprising about 210 amino acids (*D. rerio* CV2 VWD: 312–469). The C-terminal part contains a so-called C8 domain (*D. rerio* CV2 C8: 509–576) and an about 55 residues long trypsin inhibitor-like domain (*D. rerio* CV2 TIL: 590–643). In this study we have used Crossveinless 2 of the zebrafish *Danio rerio*, which shares 65% identity to the human homolog BMPER for the full-length protein and 68% amino acid sequence identity for the VWC1 domain. Analysis of the binding capabilities of the five VWC domains of CV2 showed that the N-terminal VWC domain termed VWC1 binds BMPs with high affinity, whereas the other VWC domains do not bind BMPs at all [24]. A truncation study of *D. rerio* CV2 showed that indeed only fragments containing VWC1 bind BMP-2 [35]. Furthermore, the isolated VWC1 domain and the full-length CV2 protein exhibit the identical binding affinity for BMP-2 indicating that the multidomain protein potentially has a linear architecture [24]. Thus an allosteric effect or influence on the binding mode can be ruled out and the isolated CV2 VWC1 domain can serve as a valid model for the structural and functional interaction of full-length CV2 with BMPs. For structure determination the VWC1 domain comprising residues Leu1 to Gly66 of the mature *Danio rerio* Crossveinless 2 (UniProt identifier Q5D734) was expressed as a fusion protein to thioredoxin [36]. This approach together with the use of the special *E. coli* strain Origami that has a deletion of the genes for glutathione reductase (*gor*) and thioredoxin reductase (*trxB*) allows for disulfide bond formation within the cytoplasm of *E. coli*. As the highly conserved disulfide bonds in VWC domains are important for structural stability and folding, this approach allowed production of functional protein without refolding [36]. However, the strain Origami does not carry the ahpC* mutation required in the *gor*/*trxB* double mutant background to allow reduction of essential substrates for the ribonucleotide reductase RNR [37,38] and thus Origami cells did not grow in M9 minimal medium required for isotope labeling. Thus, the plasmid encoding for the thioredoxin-VWC1 domain fusion was transformed into BL21(DE3) cells, which were then grown on M9 minimal medium supplemented with 0.5 g·L^−1^^15^N ammonium chloride and 4 g·L^−1^^13^C_6_ glucose. The temperature of bacterial cultivation was reduced to 20 °C after induction of protein expression to increase the yield of solubly expressed thioredoxin-fusion protein. After cell lysis and initial purification employing metal ion affinity chromatography (IMAC), the CV2 VWC1 protein was cleaved from thioredoxin by proteolysis using the endopeptidase thrombin. After proteolysis enzymatic activity was quenched by adding a protease inhibitor mix and the protein solution was incubated for further 72 h at 4 °C. This measure increased the yield of soluble protein in the production of the BMP receptor type IA ectodomain employing a similar thioredoxin-fusion expression approach and growing BL21(DE3) *E. coli* cells in M9 minimal medium [39]. The reducing cytoplasm of the BL21 bacterial strain in contrast to the oxidizing environment provided in *E. coli* cells of the Origami or AD494 strain results in the majority of the cysteine residues being in the reduced state rather than forming the proposed disulfide bonds. Thus immediate purification and separation from thioredoxin resulted in a decrease in protein yield. As thioredoxin can act as a disulfide isomerase and chaperone [40], it thereby increases the solubility of the fusion protein and allows for subsequent disulfide formation due to oxidation by air. After the three-day incubation thioredoxin and the CV2 VWC1 domain were separated. To obtain homogenous and active CV2 VWC1 protein a final affinity chromatography step employing a BMP-2 affinity resin was required. From one liter of M9 minimal medium about 2.5 mg pure ^13^C-,^15^N-double labeled protein could be obtained, which is about 30% of the yield obtained from expression using Luria-Miller or Terrific Broth medium. For NMR spectroscopy the protein was dialyzed against a buffer containing 20 mM sodium phosphate, 20 mM sodium chloride pH 6.6 and concentrated to 2 mM protein concentration.

### 2.2. The Distribution of the NOE-Derived Distance Restraints Suggest Flexible N- and C-Termini in the CV2 VWC1 Domain

NMR data was acquired from three samples, unlabeled containing 2 mM CV2 VWC1, one sample comprising 1.5 mM uniformly ^15^N-labeled CV2 VWC1 and one sample containing 2 mM ^13^C-,^15^N-labeled CV2 VWC1. For sequential assignment of the protein domain backbone a set of 3D triple resonance experiments comprising HN(CA)CO, HNCO, CBCA(CO)NNH, and CBCANNH were acquired, to get proton chemical shift information for side chain atoms additional 3D experiments were recorded namely HBHA(CO)NNH, H(CC)(CO)NNH-TOCSY, ^15^N-HSQC-TOCSY as well as a set of 2D homonuclear experiments, *i.e.*, DQF-COSY and TOCSY [41]. The proton chemical shifts of almost all spin systems could be assigned as well as the carbon and nitrogen chemical shifts for Cα, Cβ and carbonyl and amide atoms. 

For generation of distance restraints two NOESY-based experiments were obtained, a 3D ^15^N-HSQC NOESY and a high-resolution 2D NOESY acquired at 900 MHz. A total of 958 NOE-derived distance restraints were obtained from the NOESY spectra of which 479 are non-sequential and not intraresidue (Table 1). Additionally chemical shift restraints based on Cα and Cβ carbon chemical shifts together with the RAMA multi-dimensional torsion angle database potential term were used to improve structure calculation [42,43]. In order to avoid structural bias an extended random coil template of CV2 VWC1 exhibiting no secondary structure was used for the structure calculation. From this template 100 structures were calculated of which 10 were selected on the basis of restraints violation and total energy. Due to the small size of the domain, formation of a larger hydrophobic core or a globular structure seems very unlikely. Since in the complex bound to BMP-2 the β-sheet of CV2 VWC1 tightly interacts with the hydrophobic epitope of BMP-2 involved in type II receptor binding, it was unclear how much of the structure of the VWC domain seen in the complex is retained in the unbound state since in the unbound state these stabilizing hydrophobic interactions with the BMP ligand are lacking. However, the NOESY spectra of the free VWC1 domain of CV2 revealed a regular NOE pattern starting at residue Cys9 indicating a structured motif.

**Table 1:** Data for structure calculation and statistics for CV2 VWC1.

Distance restraints	for residues Leu1 to Gly66
NOE-derived	
Total	958
Sequential (|i-j| = 1)	479
Medium-range (|i-j| ≤ 4)	168
Long-range (|i-j| > 4)	311
Average NOE/residue	14.3
Hydrogen bonds	3
Structure statistics	
RMSD from experimental restraints (NOE) (Å)	0.067 ± 0.005
RMSD from idealized geometry	
Bonds (Å)	0.006 ± 0.001
Angles (deg.)	0.664 ± 0.053
Energies (kcal mol^.1^)	
E_total_	−82.1 ± 70.9
E_bond_	32.9 ± 6.1
E_angle_	121.1 ± 19.4
E_vdW_	47.2 ± 17.2
E_chemshift_	46.1 ± 11.1
E_ramachandran_	−522.3 ± 23.1
E_NOE_	174.0 ± 18.5
	
RMSD from average structure (Å) ^a^	
Backbone atoms for residues 8–41	0.46 ± 0.19
All heavy atoms for residues 8–41	0.92 ± 0.21
Backbone atoms for residues 1–7	1.40 ± 0.40
Backbone atoms for residues 42–66	1.99 ± 0.68
PROCHECK analysis	
Residues in most favored regions (%)	64.4 (380) ^b^
Residues in additionally allowed regions (%)	23.7 (140)
Residues in generously allowed regions (%)	11.9 (70)
Residues in disallowed regions (%)	0 (0)

^a^ An average structure was calculated with all structures superimposed onto the backbone atoms (C', Cα, N) of either residues Ser8-Glu41, Leu1-Ala7, or Lys43-Gly66, respectively. The individual structures were then aligned to the respective averaged structure using the same residue ranges as used for averaging. The r.m.s. deviations were calculated for this superposition. ^b^ The PROCHECK analysis was performed with the final structure ensemble comprising 10 selected structures. The structure ensemble contains 590 non-glycine and non-proline residues. Numbers in parenthesis mark residues in either category.

**Figure 1:**
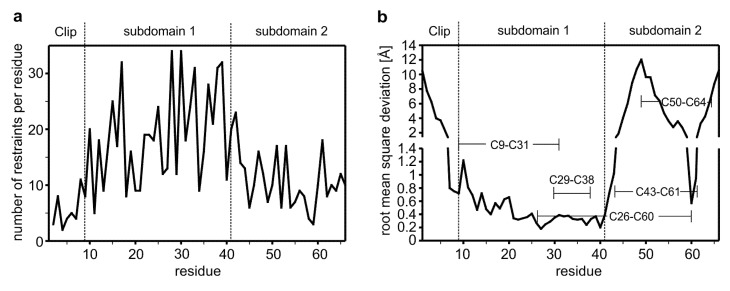
(**a**) Number of NOE-derived distance restraints per residue showing a rather low number of experimental restraints. (**b**) Root mean square deviation (r.m.s.d.) for the backbone atoms derived from a structure alignment of 10 selected structures to the average structure. Structural superposition was performed on the backbone atoms of Glu10 to Glu41). The tripartite architecture comprising the Clip segment and subdomains 1 and 2 are indicated, the disulfide bond connectivity is highlighted.

Analysis of the pattern suggests a β-strand like backbone conformation for the amino acid stretches Gly13 to Val15, Ile27 to Leu32 and Lys35 to Gln40 in the N-terminal region. In contrast in the C-terminal comprising residues Glu41 to Gly66, only in two short stretches consisting of residues Leu52 to Lys55 and Glu62 to Cys64 so-called long-range NOEs indicative for a short β-sheet segment could be observed. A comparison of the distribution of restraints shows that the N-terminal region comprising residues Cys9 to Glu41 have far more distance restraints per residue (20 NOEs per residue) than the other parts of the VWC1 domain denoting that this region/subdomain is structurally better defined. The N-terminal segment and the C-terminal part of the CV2 VWC1 domain have a similar low number of restraints per residue, *i.e.*, about eight distance restraints per residue for the N-terminal segment Leu1-Glu12 (excluding the artificially introduced tryptophan residue at the very N-terminus) and about 11 distance restraints per residue for the C-terminal region Lys42-Gly66 pointing towards a flexible or disordered structure (Figure 1).

### 2.3. The Structure of CV2 VWC1 Reveals a Modular Architecture with Flexible and Rigid Segments

**Figure 2:**
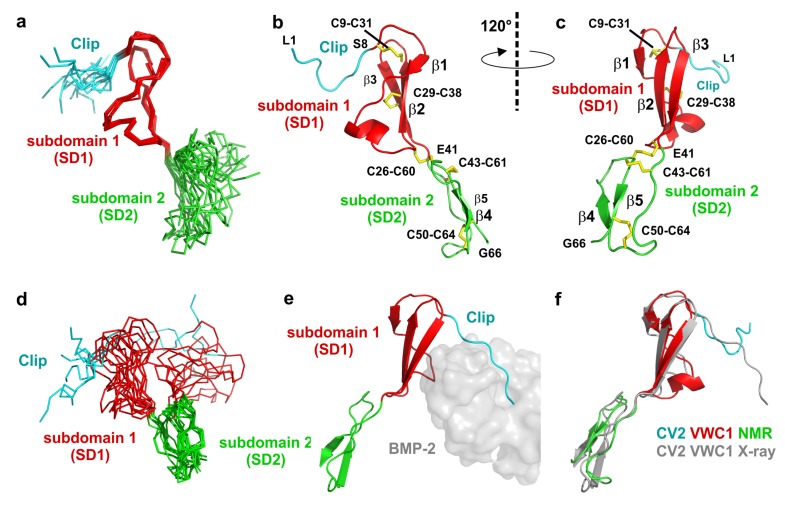
(**a**) Structure ensemble of ten NMR structures of CV2 VWC1 aligned to subdomain 1. (**b**) Ribbon plot of the NMR structure of CV2 VWC1, subdomain boundaries for the Clip segment (L1 to S8) and the subdomains 1 and 2 are indicated, disulfide bonds are shown as sticks. (**c**) As in (**b**) but rotated around the y-axis by 120°. (**d**) Structure ensemble as in (**a**) but aligned on the backbone atoms of subdomain 2. (**e**) CV2 VWC1 bound to BMP-2 (PDB entry 3BK3), the VWC1 domain is shown as ribbon plot, BMP-2 is shown as semi-transparent van der Waals surface in grey. The Clip segment folds into the BMP type I receptor-binding site, subdomain 1 is attached to the BMP type II receptor site. (**f**) Structure alignment of CV2 VWC1 in its bound (indicated in grey) and unbound conformation (labeled CV2 VWC1 NMR).

Consistent with the crystal structure of CV2 VWC1 bound to the TGF-β ligand BMP-2 (PDB entry 3BK3), structure calculation employing the NMR data yielded a similar modular architecture for CV2 VWC1 in its unbound conformation. The tripartite architecture consists of an eight residue long N-terminal segment (Leu1 to Ser8) with no regular secondary structure, which was termed Clip segment in the structure of CV2 VWC1 bound to BMP-2. Attached to this, the NMR structure presents a 32 residue sized structured domain (Cys9 to Glu41), which was termed subdomain 1 (SD1) previously. This subdomain 1 mainly consists of a relatively flat, all-antiparallel three-stranded β-sheet with one short and two longer β-strands. The C-terminal part that has been named subdomain 2 (SD2 running from Lys42 to Gly66) forms a ring-like structure with the C-terminus folded on top of this ring. The protein backbone of the subdomain 2 does not adopt a regular secondary structure and is less compact than subdomain 1 (Figure 2). In its bound conformation the VWC1 Clip segment tightly interacts with the type I receptor binding epitope of the ligand BMP-2 thereby adopting a rigid conformation as is visible from the low temperature factors of these residues, which are on the same level as residues in the highly structured subdomain 1. In the NMR structure a large part of the N-terminal Clip segment shares no direct contact with residues from subdomain 1 and 2 and thus is expected to be highly flexible. Indeed very few sequential interresidue NOEs are observed for this segment comprising Leu1 to Glu6 consistent with the hypothesis that this segment is flexible and disordered in the unbound state. Conformingly, a superposition of the structure ensemble of 10 structures shows that the N-terminal stretch Leu1 to Ala7 exhibits a high r.m.s. deviation of 1.4 Å for the Cα atoms (compared to the average structure) whereas the Cα atoms of subdomain 1 comprising residues Ser8 to Glu41 display a low r.m.s. deviation of 0.5 Å (Figure 1b and Figure 2a–c). Thus the N-terminal Clip adopts its final conformation only upon complex formation with its BMP ligand-binding partner and might be thus adaptable to different interaction partners to broaden ligand specificity. Interestingly, analysis of the structure ensemble revealed that subdomain 2 of CV2 VWC1 is also highly disordered as documented by the high r.m.s. deviation of 2 Å for the Cα-position of residues Lys42 to Gly66 (Figure 1b and Figure 2a,d). At first sight this is unexpected, as in the structure of CV2 VWC1 bound to BMP-2 the temperature factors of a stretch of backbone atoms in subdomain 2 (*i.e.*, Ala51 to Lys55) are among the lowest observed in this crystal structure. Furthermore this small subdomain of 27 amino acids harbors three disulfide bonds (Cys26-Cys60, Cys43-Cys61 and Cys50-Cys64) not only linking subdomain 2 to subdomain 1 but also forming a disulfide network potentially stabilizing and fixating this subdomain. 

However, in the structure of the unbound state of CV2 VWC1 only a low average number of NOE distance restraints per residue (11 restraints per residue) have been found for this segment. In addition no regular NOE pattern indicative for larger secondary structure elements were observed. Only two short stretches, Ala51-Val54 and Lys63-Gly66, which cross over, might potentially form a two-stranded β-sheet if subdomain 2 becomes stabilized upon interaction with a binding partner (Figure 2a–d). So how can the discrepancy between a highly structured and highly flexible subdomain 2 seen in the crystal and NMR structure, respectively, be explained? Firstly, in the crystal structure the subdomain 2 of CV2 VWC1 does not contact the ligand BMP-2 at all and hence deletion mutant constructs of the VWC1 domain containing only the Clip segment and subdomain 1 bound BMP-2 with identical affinity as the full-length VWC1 domain (see also Figure 2e) [23]. Secondly, inspection of the crystal lattice arrangement of the CV2 VWC1-BMP-2 complex reveals that the first β-strand of subdomain 2 (*i.e.*, Ala51 to Val54) engages in a hydrogen-bond network with the so-called pre-helix loop (Pro50 to Asp53) of a lattice-related BMP-2 molecule thereby stabilizing and fixing the structure and orientation of subdomain 2 with respect to the other regions of the CV2 VWC1 moiety.

The observation of a disordered subdomain 2 in the N-terminal von Willebrand type C domain of CV2 is also consistent with the structure of the Chordin-like cysteine-rich repeat (which represents a VWC domain) of procollagen IIA [44]. Like our NMR structure of CV2 VWC1 presented here the VWC module of procollagen IIA displays a highly flexible N-terminus and a disordered subdomain 2 suggesting that the structures of VWC domains are generally following this architecture. The structural flexibility of the subdomain 2 of CV2 VWC1 and the fact that it is dispensable for BMP-2 binding raised the question of its functional importance. It was suggested that the subdomain 2 of CV2 VWC1 might present a docking/interaction site for other interaction partners [23], possibly the BMP modulator proteins Chordin and Twisted Gastrulation (Tsg), which have been shown to bind to Crossveinless 2 [45]. For the VWC domains of Chordin and Chordin-like 2 (CHL2) a study, however, showed that interactions between these Chordin-related factors and Tsg seem to occur exclusively via the subdomain 1 [46], but structure data for the latter VWC modules are not known and sequence homology between the VWC1 of CV2 and those of Chordin/CHL2 is low. Recently, Zhang *et al.* observed that the subdomain 2 of CV2 VWC1 is part of a larger composite epitope and crucial for the interaction of Crossveinless 2 and Chordin [35]. Thus the subdomain 2 of the VWC module can exert a functional role and by interaction with other binding partners its flexible structure might become rigid.

### 2.4. The Clip Segment Must be Pre-Oriented for High Affinity Binding to BMPs

The high affinity binding of the quite small VWC domain 1 of CV2 to BMP-2 in the low nanomolar range has raised the question how this binding strength can be achieved from a combination of a small number of hydrogen bond interactions and a small-sized hydrophobic epitope. Mutagenesis and *in vitro* binding analysis have shown that the tight BMP-2 binding strongly depends on the hydrophobic patch formed by residues Ile21 located in the β1β2 loop, Ile27 located in β-strand 2 and the disulfide bond formed between Cys29 and Cys38 “crosslinking” β-strands 2 and 3 of CV2 VWC1 [23]. Mutation of either one of the two isoleucine residues attenuates the overall binding affinity 10-fold. Surprisingly, deletion of the N-terminal Clip, e.g., either by removing residues Leu1 to Thr5 or Leu1 to Ala8 reduced binding affinity far more drastically by 1,135 and 2,400-fold [23], although the Clip segment presents only a single peptide motif. Five hydrogen bonds and one hydrophobic knob-into-hole interaction between the CV2 VWC1 Clip segment and BMP-2 have been observed and individual mutation of the residues involved in these polar bonds, e.g., T3P and T5P, or the hydrophobic interaction, e.g., I2A, show that each of these interactions contribute to the overall affinity although the large effect of the Clip segment truncation mutations is not explained by simply adding the loss of binding observed for these single amino acid exchanges [23]. From functional analyses it seems that the interaction of CV2 VWC1 with BMP-2 is dominated by the interaction of the linear Clip segment with the type I receptor epitope of BMP-2. While this is already difficult to explain on the basis of the structure of the complex of CV2 VWC1 bound to BMP-2, it is even harder to conceive this observation if we consider that before complex formation this Clip segment is highly flexible and disordered as provided by the NMR structure analysis.

Interestingly, although the N-terminal part of the Clip segment is disordered, its general orientation seems prefixed (Figure 2). N-terminal of the first β-strand (Gly13 to Val15) the loop leading to the Clip segment makes a sharp turn such that the C-terminal residues of the Clip fold over the β-sheet of the subdomain 1. For the methyl group of alanine 7 of the Clip segment, which is located at the end of the turn, we could observe NOEs to the methyl groups of alanine 36 and isoleucine 18 indicating that the N-terminal Clip folds into a structure somewhat resembling the bound conformation even in the absence of the BMP ligand (Figure 2f). This results in a preformed hook-like architecture of Clip-subdomain 1 module, which upon binding to BMPs brings the N-terminal Clip already in close proximity even if the final local interactions, *i.e.*, the hydrogen bond network and knob-into-hole interactions, between the Clip and the type I receptor binding site of BMPs are not yet formed. This mechanism likely lowers the entropy cost that would be otherwise required to fully fold a completely disoriented and flexible Clip segment into the final BMP epitope and explains the high cooperativity and large contribution of the Clip segment to the overall binding energy to bind to BMP-2 [23].

**Figure 3:**
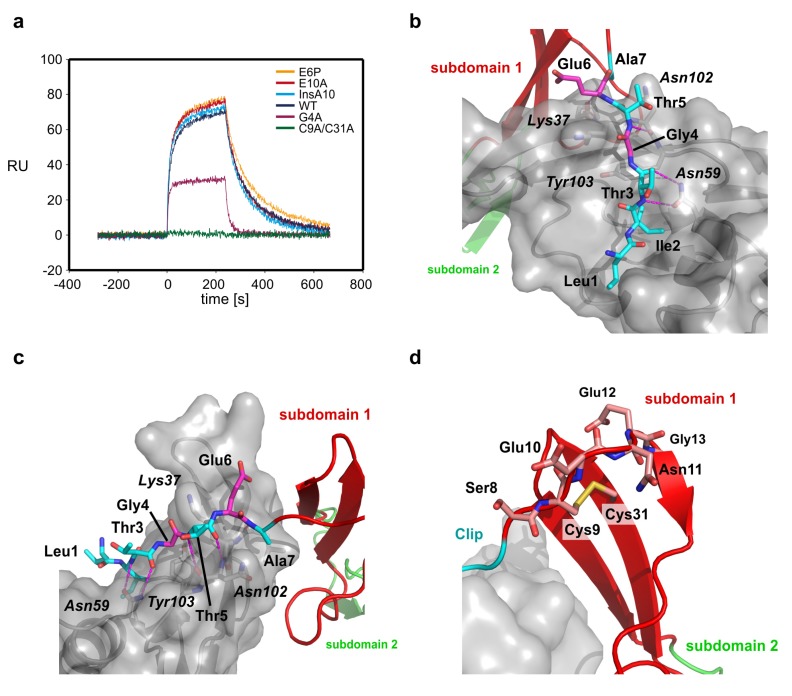
(**a**) Sensorgram of the interaction of CV2 VWC1 and mutants thereof. For comparison the perfusion of CV2 VWC1 proteins with an analyte concentration of 75 nM is shown. Perfusion of the analyte starts at time point 0, after 240 s analyte perfusion is stopped and dissociation data is observed from perfusion of the biosensor with buffer. Whereas the mutants E6P, E10A and the CV2 VWC1 mutant harboring an alanine insertion at position 10 display wildtype like binding kinetics, mutants G4A and the disulfide bond deletion mutant C9A/C31A show a dramatic loss of binding and an altered binding kinetics. (**b**) Details of the interaction between the residues of the CV2 VWC1 Clip segment and the binding site of BMP-2. Intermolecular hydrogen bonds are shown as stippled lines. The effect of mutating Leu1, Ile2, Thr3 and Thr5 (marked with cyan carbon atoms) has been investigated before, mutating Gly4 and Glu6 (carbon atoms colored in magenta) has different effect on BMP-2 binding. Whereas mutation E6P does not alter BMP-2 affinity, exchange of Gly4 by alanine dramatically lowers BMP-2 binding affinity likely due to introducing steric hindrance between the side chain at position 4 of the Clip and residues, e.g., Tyr103 at the BMP-2 surface. (**c**) As in (**b**) but seen from a different orientation. (**d**) The disulfide bind between Cys9 and Cys31 fixes the orientation of the Clip segment by anchoring the Clip segment onto the β-sheet of subdomain 1 and introducing a hook-like structure between residues Glu10 to Gly13. Introducing a single additional residue (mutant InsA10) at the C-terminal site of Cys9 seems not alter the hook-like structure enough to disrupt BMP-2 binding.

With this hypothesis in mind it is still unclear what leads to the formation of the turn to pre-orient the Clip segment. We have thus performed a functional study using surface plasmon resonance (SPR) to unravel the residues involved in Clip pre-orientation and fixation (Table 2, Figure 3a). First we exchange two glutamate residues in the N-terminal segment, Glu6 and Glu10. The first, Glu6 is located in close proximity to a lysine residue in subdomain 1 of CV2 VWC1, Lys35, and might thus orient the N-terminal Clip through electrostatic interactions between the two residues (Figure 3b). We choose to exchange Glu6 by a proline to indirectly test also the contribution of the neighboring residue Ala7. The amide group of Ala7 is involved in a hydrogen bond with the carbonyl group of Leu100 of BMP-2 and introducing the proline ring structure alters the backbone conformation of the Glu6-Ala7 peptide bond thereby disrupting this intermolecular hydrogen bond. However, SPR measurements showed that CV2 VWC1 E6P binds BMP-2 with wildtype-like affinity [K_D_(wt) 14 nM; K_D_(E6P) 12 nM] and also does not exhibit a different binding kinetics (Table 2, Figure 3a).

**Table 2:** *In vitro* interaction data for the binding of CV2 VWC1 and mutants thereof to BMP-2.

CV2 VWC protein	K_D_ (eq) (nM)	*k*_on_ × 10^5^ (M^−1^s^−1^)	*k*_off_ × 10^−3^ (s^−1^)	K_D_ ( *k*_off_/*k*_on_) (nM)	K_D_ (var)/K_D_ (wildtype)
wildtype	-	6.5 ± 0.2	8.7 ± 0.1	13 ± 4	1
G4A	239 ± 44	-	≥50	-	18.4
E6P	-	6.3 ± 0.2	7.3 ± 0.1	12 ± 3.6	0.9
E10A	-	7.5 ± 0.3	9.5 ± 0.1	13 ± 4	1
InsA10	-	9.1 ± 0.2	1.2 ± 0.01	13 ± 4.4	1
C9A/C31A	≥3300 ± 1400	-	≥50	-	255

The glutamate residue at position 10 in CV2 VWC1 is located in the turn and is highly conserved in the CV2 VWC1 domain of different species indicating a functional role, although its side chain is oriented towards the solvent and in the complex does not make any contact with the BMP ligand. Exchange of Glu10 to alanine did not alter the binding affinity to BMP-2 (K_D_(E10A) 13 nM) and suggests that this residue is not involved in orienting the Clip segment (Figure 3c,d). The flexibility of the Clip segment might also contribute to adaptability to different BMP ligand binding partners. So far only the importance of the residues involved in the formation of the hydrogen bond network, Thr3 and Thr5 and the residue Ile2 that make a hydrophobic interaction, all of which are highly conserved in VWC1 domains of CV2 of different species, has been analyzed (Figure 3b). The glycine residue at position 4 is also highly conserved in different CV2 VWC1 and its Cα-atom is placed such that the side chain of a different amino acid would be pointing towards the surface of BMP-2 thus requiring a displacement of the peptide backbone of the N-terminal Clip segment (Figure 3b). Thus mutation of Gly4 would potentially reveal how adaptable the conformation of the Clip segment is to allow for tight binding to different TGF-β/BMP ligands. Exchange of Gly4 by alanine showed that small displacement of the Clip backbone as introduced by the addition of a methyl group onto the backbone leads to a drastic loss in BMP interaction. The mutant CV2 VWC1 G4A exhibits a significantly altered binding kinetics as most evident from a much faster dissociation rate constant (*k*_off_~6 × 10^−2^ s^−1^ compared to *k*_off_(wt)~9 × 10^−3^ s^−1^) (Table 2, Figure 3a). Due to the fast binding kinetics the affinity was determined from the dose-dependency of the equilibrium binding yielding an affinity of about 240 nM more than 17-fold lower as observed for the interaction of wildtype CV2 VWC1 with BMP-2. Thus for high-affinity interaction of BMP-2 with CV2 VWC1 the Clip segment has to tightly fit into the cleft located in epitope of BMP-2.

To test the possible origin of the Clip segment pre-orientation we analyzed the influence of the disulfide bond between Cys9 and Cys31. Cysteine 9 is placed at the N-terminal end of the turn leading into the short first β-strand 1 of CV2 VWC1 and thus likely has a key role in placing the N-terminal Clip segment. From structure analysis it seems that only due to the direction of the first β-strand in VWC1 and Cys9 located ahead of the turn-like structure and being engaged in the disulfide bond with Cys31, which is located beneath in β-strand 2, the turn-like structure is initiated resulting in the Clip segment to be oriented almost parallel towards subdomain 1 and running into the opposite direction thus forming this paperclip- or hook-like architecture. Thus disrupting this disulfide bond should have a dramatic effect on placement of the Clip segment and due to the importance of the latter for BMP binding abolish interaction with BMP (Figure 3d). 

We therefore prepared the double mutant CV2 VWC1 C9A/C31A, which could be expressed, refolded and purified similar to wildtype CV2 VWC1 indicating that the fold of the mutant protein is not grossly altered. SPR analysis shows that BMP-2 binding is almost completely abolished by this mutation estimating the affinity to 3 µM or less (Figure 3a,d). This low residual affinity of CV2 VWC1 C9A/C31A is close to the values found for the VWC1 mutants with a truncated Clip segment [23] and thus confirms that the conserved disulfide bond between the first and the fourth cysteine residue is the key element for pre-orienting the N-terminal Clip segment in CV2 VWC1. It is interesting to note that cleavage of this disulfide bond would present a potential mechanism to regulate BMP binding by CV2. Accordingly, Ganderton *et al.* have recently described that the disulfide bond between Cys2431 and Cys2453 in the von Willebrand factor (VWF) C2 domain, which is homologous to the CV2 VWC1 disulfide Cys9-Cys31, occurs in a mixed redox status with about 75% of the disulfide bond being reduced and thereby allowing for reshuffling of the disulfide bond connectivity and possibly enabling VWF fiber formation [47]. However, the extracellular reductase required for specific cleavage of the disulfide bond yet needs to be identified. 

Besides the disulfide bond itself also the loop conformation and length between the first cysteine Cys9 and β-strand 1 can influence the orientation of the N-terminal Clip segment. Sequence comparison of different VWC modules of different BMP modulator proteins shows that many VWC domains indeed contain an amino acid insertion of 2 to 10 residue length in the β1β2-loop, which might alter the orientation of the Clip segment. We thus tested whether insertion of an alanine residue between Cys9 and Glu10 would alter the position of Cys9 thereby also affecting the Clip segment. However, the mutant CV2 VWC1 InsA10 exhibits a wildtype-like affinity for BMP-2 and also the binding kinetics are unaltered suggesting that insertion of a single residue in the β1β2-loop length have no effect on BMP binding (Table 2, Figure 3d).

### 2.5. The First VWC Domain of Crossveinless 2 Possibly Unique Compared to Other VWC Domains

The von Willebrand type C domain is found in all members of the Chordin superfamily as well as other BMP modulator proteins, but also in proteins that do not interact with BMPs at all [26,48,49]. As mentioned above, even for BMP modulator proteins with multiple VWC domains, BMP binding capability can vastly vary between the different VWC modules. The small size of the VWC domain of only 55 to 60 amino acid residues on the one hand and the—frequently—high binding affinity to BMP ligands on the other hand make the mechanism by which the von Willebrand type C domain recognizes and interacts with BMPs an enigma. Sequence analysis of BMP-binding and BMP non-interacting VWC domains have yet failed to provide a code for understanding the underlying protein-protein recognition. The crystal structure analysis of the CV2 VWC1 domain bound to BMP-2 has provided some insight into how VWC domains might bind their interaction partners [23]. Functional analyses have shown that only the N-terminal Clip segment and the small subdomain 1 of the tripartite VWC structure is required for high-affinity BMP-2 binding. Furthermore, deletion studies suggested that the Clip segment, despite being only a short peptide segment and showing little interaction between its amino acid side chains and BMP-2, is absolutely crucial for BMP binding. Due to its linear nature it has been assumed that in the unbound state the Clip peptide of CV2 VWC1 would be likely flexible and disordered, determination of the free unbound conformation by NMR spectroscopy has now added valuable information for the understanding of the protein-protein recognition between these two proteins. The pre-defined orientation of the Clip certainly explains how the peptide can contribute significantly to the overall binding energy. With the knowledge of the key elements for high-affinity BMP binding comprising a pre-oriented Clip and a hydrophobic patch in the small subdomain 1 do we have a general recognition mechanism for the von Willebrand type C domains at hand? Sequence analyses of VWC domains of different BMP modulator proteins show that the different VWC domains, even if we select only those experimentally shown to bind to BMPs, have very little sequence conservation. Many VWC domains seem to have no sequence homologous to the CV2 VWC1 Clip segment or have either amino acid insertions or a highly different amino acid sequence in the subdomain 1 [46]. 

**Figure 4:**
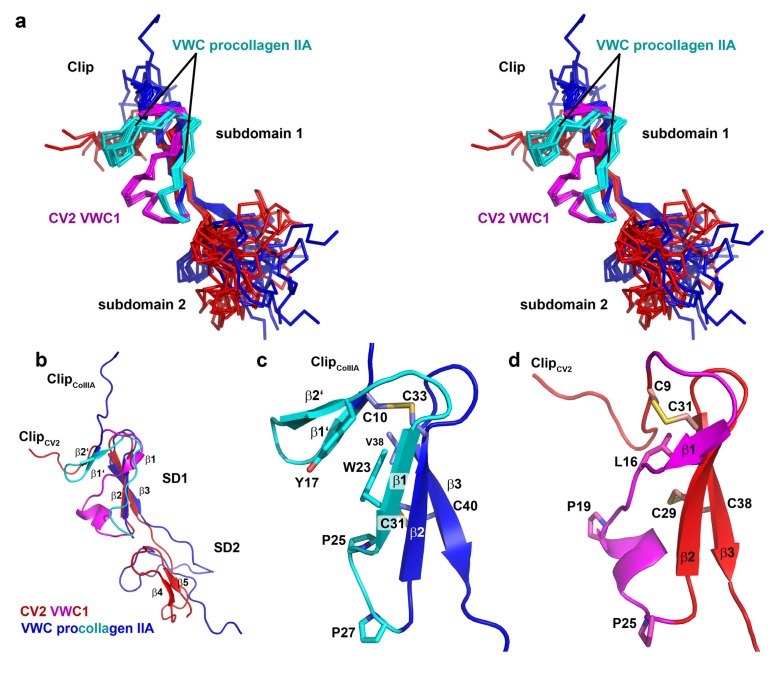
(**a**) Stereoview of a superposition for the structure ensembles (10 structures each) of CV2 VWC1 (red, differing elements in magenta) and procollagen IIA VWC (blue, differing elements colored in cyan). (**b**) Ribbon plot of CV2 VWC1 structurally aligned to the VWC domain of procollagen IIA. (**c**) Magnification of the VWC subdomain 1 of procollagen IIA highlighting the additional two-stranded β-sheet 1' and 2' which are possibly formed (and stabilized) due to hydrophobic interaction between residues in the β-strands β1' and β2' and β1. (**d**) Magnification as in (**c**) but for the VWC1 domain of CV2. At the C-terminal side of the first cysteine the loop adopts a turn-like structure with a short β-strand 1 and merging into a one-turn helix. One clear difference is in the length of the peptide sequence interspersed between the two conserved proline residues (P25 and P27 in VWC procollagen IIA and P19 and P25 in CV2 VWC1).

**Figure 5:**
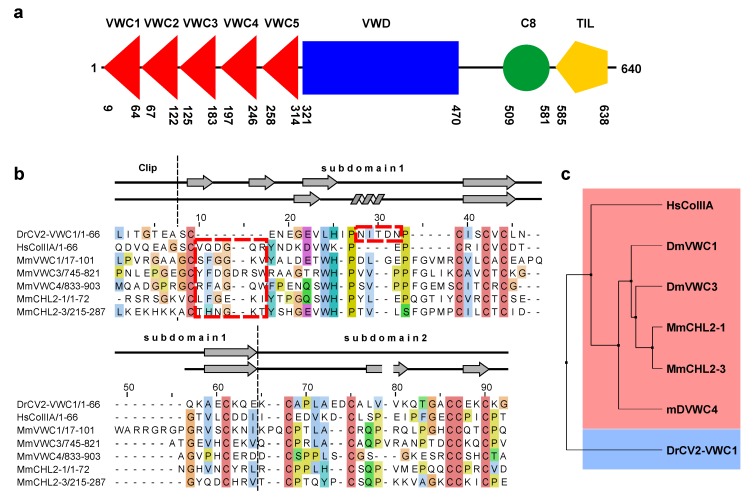
(**a**) Architecture of the BMP modulator Crossveinless 2. The VWC domains 1–5 are indicated by red triangles, the VWD domain by a blue rectangle, the C8 and the TIL domain by a green circle and an orange pentagon. The amino acid boundaries of the protein domains are obtained from the PFAM and SMART database using the amino acid sequence of mature *Danio rerio* Crossveinless 2. (**b**) Sequence alignment based on a structure comparison of the VWC domains of *Danio rerio* CV2 (VWC1) and human procollagen IIA (UniProt code P02458) showing that VWC domains might cluster into two classes. For comparison other VWC domains from modulator proteins of the Chordin family are shown (MmVWC1, 3, 4: *Mus musculus* Chordin VWC domains 1, 3 and 4, UniProt code Q9Z0E2; MmCHL2-1, 3: *Mus musculus* Chordin-like 2 VWC domains 1 and 3, UniProt code Q8VEA6). Architectural features of the CV2 VWC1 domain are indicated; secondary structure elements as present in the NMR structures of procollagen IIA VWC (upper line) and CV2 VWC1 (lower line) are shown. The boxes marked by red stippled lines indicate the two discriminatory sequence features leading to the differing structures observed for both VWC domains. (**c**) Phylogenetic analysis using the sequence alignment shown in (**a**).

So far only two structures are available for the VWC domains of CV2 and procollagen IIA [44] and their comparison shows rather large differences in the region involved in binding to BMPs (Figure 4a,b). Most evident, the important Clip segment points into opposite directions in both VWC domains, with the Clip folding back onto the subdomain 1 in CV2 VWC1 thereby forming the paperclip or hook-like architecture, whereas in the procollagen IIA VWC module the Clip folds away from the subdomain 1 potentially enforcing a more linear structure (Figure 4a,b) as has been proposed for the consecutive VWC domains of the von Willebrand Factor on the basis of high resolution electron microscopy and multiple particle class averaging [50]. In addition, the subdomain 1 also differs significantly between the two VWC domains. In CV2 VWC1 after the Clip segment with the disulfide Cys9-Cys31 the subdomain 1 starts with a short three-residue β-strand, which merges into a loop containing a single turn before ending in the conserved two-stranded β-sheet (Figure 4b,d). In the VWC module of procollagen IIA with the Clip arriving from the top an additional small two-stranded β-sheet is present before the peptide chain merges into a longer β-strand 1 and the conserved two-stranded central β-sheet characteristic for the VWC subdomain 1 (Figure 4b,c) [44]. It is interesting to note that despite an equivalent disulfide bridge formed by Cys9 and Cys31 in CV2 VWC1 and by Cys10 and Cys33 in the VWC domain of procollagen IIA, the structures before and following the first cysteine residue differ significantly. The detailed comparison of both VWC modules above further suggests that the amino acid sequence of the first loop of the subdomain 1 might be in a different register in both structures, with the residues forming the additional two-stranded β-sheet in the VWC of procollagen IIA being “shifted” into the longer β1β2-loop forming a helical turn. Thus, the VWC1 domain of Crossveinless 2 seems structurally different from the VWC module of procollagen IIA and thus there might be at least two structurally distinct classes of VWC domains. The sequence alignment of both structurally known VWC domains together with a set of other VWC domains of BMP modulator proteins experimentally shown to bind to BMP-2 indeed shows that two sequence elements separate the listed VWC domains into either one of two classes (Figure 5b). 

A six-residue insertion after the first cysteine residue with a central glycine residue allows for the formation of the additional two-stranded β-sheet (β1’ and β2’) as seen in the VWC module of procollagen IIA (Figure 5b). In addition, these procollagen IIA-like VWC domains contain a conserved tyrosine and a tryptophan residue, which build a small hydrophobic core stabilizing the β1’β2’ β-sheet (Figure 4c). In the VWC1 domain of CV2 these additional residues are missing and thus the shorter segment can only form a turn-like structure (Figure 4d). In contrast, the VWC1 domain of CV2 has a five-residue insertion between the two highly conserved proline residues (Pro19 and Pro25 of CV2 VWC1), which fold into a single turn helix (Figure 5b) not seen in the VWC module of procollagen IIA. With these two key elements enabling us to discriminate the two classes, the other structurally not yet characterized VWC domains of Chordin and Chordin-like 2, which are known to bind to BMPs, seem to belong to the procollagen IIA-like VWC class rather than to the CV2 VWC1-like class. A phylogenetic analysis using this sequence alignment indeed shows that CV2 VWC1 and procollagen IIA VWC are most distantly related, whereas the other VWC domains cluster with the VWC domain of procollagen IIA (Figure 5c).

Thus what we have learned about the molecular recognition of BMP-2 by CV2 VWC1 from the crystal structure of CV2 VWC1 in complex with BMP-2 and the NMR structure of unbound CV2 VWC1 might only apply partially to other VWC domains. The different conformations within subdomain 1 and the Clip segment in the two structurally characterized VWC domains clearly indicate that the binding of VWC to BMPs is a very complex mechanism and to be able to predict the mode of BMP:VWC interaction still more structural data on different VWC is required.

## 3. Experimental

### 3.1. Expression and Purification of the VWC1 Domain of Crossveinless 2

For bacterial production of recombinant CV2 VWC1 protein cDNA encoding for zebrafish (*Danio rerio*) CV2 VWC1 was cloned into a modified pET32a (Novagen, Darmstadt, Germany) expression vector using the *NcoI/BlpI* restriction sites. Using this vector results in expression of a fusion-protein with a N-terminal thioredoxin- with an attached His_6_-Tag, both of which can be removed by proteolysis using thrombin. Mutants of CV2 VWC1 were obtained from two-step PCR mutagenesis. For expression of unlabeled proteins the *E. coli* strain Origami B (DE3) was used and transformed cells were selected by adding 100 µg mL^−1^ ampicillin, 15 µg mL^−1^ kanamycin and 12.5 µg mL^−1^ tetracycline. Transformed cells were grown in LB medium (Melford, Chelsworth, UK) at 37 °C. At an optical cell density (OD_600_) of 0.4 cells were cooled to 18 °C and growth was continued for 30 min before protein expression was induced for 18 h by addition of 1 mM isopropyl-1-thio-β-D-galactopyranoside (IPTG). Cells were harvested by centrifugation (6,000 × g, 15 min, 4 °C), and washed with TBSE buffer (10 mM Tris-HCl pH 7.4, 150 mM NaCl, 2 mM EDTA). To extract the soluble fusion-protein 7.5 g cells were resuspended in 20 mM Tris-HCl pH 8.0, 500 mM NaCl, 10 mM imidazole and lysed on ice by sonication (HD 3200, Bandelin Sonoplus, Berlin, Germany, 20 min, 55% amplitude). Cell debris was removed by centrifugation (25,000 ×g, 30 min, 4 °C) and the protein was purified from the clarified supernatant using immobilized metal ion affinity chromatography (IMAC) (HisTrap FF, 5 mL, GE Healthcare, Munich, Germany). The fusion-protein was eluted by application of 20 mM Tris-HCl pH 8.0, 500 mM NaCl, 500 mM imidazole and protein-containing fractions were pooled and dialyzed overnight to remove imidazole. To cleave off the thioredoxin-His_6_ fusion part proteolysis was performed using 0.15 U thrombin (Sigma, Munich, Germany) per 1mg fusion protein at 30 °C for 4 h. Protease activity was quenched by adding protease inhibitor (protease inhibitor cocktail mix III, Calbiochem, Darmstadt, Germany). After dialysis against 20 mM Tris pH 7.5 the protein mixture containing thioredoxin and CV2 VWC1 was incubated for three days at 4 °C to increase yield. To separate CV2 VWC1 and thioredoxin proteins anion exchange chromatography was performed (HiTrap Q Sepharose FF, 5 mL, GE Healthcare) employing a gradient from 0 to 250 mM NaCl. For final purification a reversed phase HPLC (Jupiter C4, 10 µm, 250 × 10 mm, Phenomenex, Aschaffenburg, Germany) and a 0.1% trifluoroacetic acid-acetonitrile gradient was used.

To obtain uniformely ^15^N- and ^13^C-, ^15^N-labeled CV2 VWC1 protein expression was performed using M9 minimal medium supplemented with 0.5 g L^−1^^15^NH_4_Cl and 8 g L^−1^ glucose or 4 g L^−1^^13^C_6_-glucose (Cambridge Isotope Labeling CIL, Saarbrücken, Germany) [51]. As the strain Origami B does not grow on M9 minimal medium, the expression plasmid encoding for the thioredoxin-CV2 VWC1 fusion protein was transformed into the *E. coli* strain BL21 (DE3), which does not allow for cytoplasmic disulfide bond formation and thus made the incubation step after thrombin proteolysis an essential prerequisite. Isolation and purification of the CV2 VWC1 protein followed the protocols as described above.

### 3.2. NMR Data Acquisition and Analysis

For NMR analysis CV2 VWC1 proteins were dissolved in concentrations 1.5 to 2 mM in 20 mM sodium phosphate, 20 mM sodium chloride pH 6.6 supplemented with 5% (v/v) D_2_O and 0.2% (w/v) sodium azide. NMR data were recorded at 27 °C employing Bruker Avance spectrometers operating either at 600 or 900 MHz proton frequencies and using triple-resonance, triple-axis gradient cryoprobes. Data were processed using the software TopSpin version 2 and analyzed using Aurelia version 3.1 (Bruker BioSpin GmbH, Rheinstetten, Germany). Backbone sequential assignments were derived from a set of triple resonance 3D experiments, namely CBCA(CO)NNH, CBCANNH, HN(CA)CO and HNCO [41]. Side chain assignments were obtained from a triple-resonance 3D H(CC)(CO)NNH-TOCSY as well as a 3D ^15^N-filtered HSQC-TOCSY experiment, the latter of which was acquired at 900 MHz proton frequencies. In the triple-resonance 3D experiments in both indirect dimensions 96 complex points were measured, which during processing were extended by zero filling to 256 points for indirect carbon and nitrogen dimensions and by linear forward prediction for indirect proton dimensions. For the ^15^N-isotope-filtered experiments 220 complex data points were acquired in the indirect proton dimension, which was extended by linear forward prediction to 256 points during processing. Additional proton chemical shifts were derived from high-resolution 2D DQY-COSY (8192 points in the direct dimension, 1024 points acquired in the indirect dimension) and 2D TOCSY (4096 points in the direct dimension, 1024 points acquired in the indirect dimension) experiments acquired at 900 MHz proton frequencies. Carbon chemical shift assignments were complete for Cα, Cβ and carbonyl carbon atoms for residues Leu1 to Gly66, similarly almost all proton chemical shifts could assigned using this set of 2D and 3D NMR spectra. Distance data were obtained from a 3D ^15^N-HSQC-NOESY spectra (mixing time 80 ms) and a high-resolution 2D NOESY (mixing time 80 ms) both acquired at 900 MHz proton frequencies. NOESY crosspeaks were converted into distances by dividing crosspeak intensities into three classes very strong, medium and weak resulting in distance restraints with upper boundaries of 2.5, 3.5 and 5.0 Å, respectively. For the use of pseudo-atoms to handle protons with degenerate chemical shifts, e.g., of methyl or methylene groups or aromatic ring protons, additional upper limit boundaries were added. Structure calculation was performed using XPLOR NIH version 2.21 [52] employing a simulated annealing protocol and starting from a linear structure template [53]. In addition to the NOE derived distance restraints, chemical shifts obtained for Cα and Cβ carbon atoms were employed making use of the chemical shift potential [54], to further improve the quality of the structures the conformational database potential was used during final stages of structure calculations [43]. For the final coordinate ensemble, 100 structures were calculated of which 10 were selected on the basis of lowest restraint violations.

### 3.3. *In Vitro* Interaction Analysis Using Surface Plasmon Resonance

All *in vitro* interaction analyses employing surface plasmon resonance (SPR) were performed using a ProteON XPR36 system (Bio-Rad Laboratories Inc., Technion, Haifa, Israel). All measurements were performed at 25 °C using HBST_500_ (10 mM HEPES pH 7.4, 500 mM NaCl, 0.005% Tween20, Sigma) as running buffer. For the interaction study BMP-2 was immobilized as ligand via the so-called amino-coupling methodology and the CV2 VWC1 proteins were used as analytes. For immobilization the alginate matrix of two flow cells of a ProteON GLC-Chip was activated for 120 s using a mixture of 75 mM 1-ethyl-3-[3-dimethylaminopropyl]carbodiimide hydrochloride (EDC, Bio-Rad Laboratories Inc., Munich, Germany) and 18.8 mM N-hydroxysulfosuccinimide (Sulfo-NHS, Bio-Rad Laboratories Inc.) in H_2_O at a flowrate of 30 µL min^−1^. To act as reference cell the activated matrix of one flow cell was then inactivated with 1 M ethanolamine pH 8.5 (Bio-Rad Laboratories Inc.). On the second flow cell *E. coli* derived recombinant human BMP-2 [55] was immobilized by perfusing (25 µL min^−1^) a solution of 600 nM BMP2 in 10 mM sodium acetate pH 4.0. When the density of immobilized BMP-2 reached a level between 1000 and 2000 RU (1 RU = 1 pg mm^−2^) immobilization was stopped and residual activated carboxyl-groups at the alginate polymer surface were inactivated similarly as above by perfusing 1 M ethanolamine for 300 s (30 µL min^−1^) over the biosensor.

For acquisition of *in vitro* interaction data wildtype CV2 VWC1 proteins and mutants thereof were dissolved in HBST_500_ buffer. The protein solutions were then perfused in six different analyte concentrations usually ranging between 0.1 to 10 times the equilibrium-binding constant over the biosensor surface. Perfusion was performed for 240 s at a flow rate of 100 µL·min^−1^ to acquire data for the association. For dissociation data, perfusion was switched to HBST_500_ buffer only (flow rate 100 µL·min^−1^) and the SPR signal was monitored for 360 s. For regeneration of the BMP-2 biosensor the surface was flushed with two short injections of 4 M MgCl_2_ lasting 18 s. SPR data was analyzed using the software ProteOn Manager version 3.06 (Bio-Rad Laboratories Inc.). Sensorgrams were fitted globally employing a Langmuir 1:1 interaction model, parameters for association rate constant *k*_on_, dissociation rate constant *k*_off_ and maximal SPR signal RU_max_ were fitted individually for the six analyte concentrations. The equilibrium binding constant was derived from the equation K_D_ = *k*_off_/*k*_on_, for CV2 VWC1 mutants binding weakly or with a binding kinetics exceeding *k*_on_ > 10^6^ M^−1^s^−1^ or *k*_off_ > 5 × 10^−2^ s^−1^ instead using the kinetic parameter to derive K_D_ the dose-dependency of equilibrium binding was analyzed. Standard deviations of K_D_ values were derived from the standard deviations of the rate constant values obtained from the individual measurements using six different analyte concentrations. All K_D_ values are considered apparent equilibrium binding constants due to the experimental conditions and might differ from binding affinities derived by other methods.

## 4. Conclusions

BMP modulator proteins exert important roles *in vivo* by tightly regulating the local activity BMP and TGF-β ligands. Without their antagonistic activity the functionality of BMPs as morphogens would likely be impossible. Due to their small size and high sequence diversity the molecular recognition and binding mechanism of VWC domains serving as BMP binding modules in BMP modulator proteins of the Chordin/Chordin-related and other families has remained an enigma. A structure-/function study of the complex of the VWC1 domain of Crossveinless 2 bound to BMP-2 showed that the tight binding is based on the highly cooperative interaction of the N-terminus of the VWC domain, termed Clip, and a small subdomain of about 30 residues in size, termed subdomain 1 [23]. In particular the large contribution of the linear peptide of the Clip segment to the overall binding energy seemed puzzling assuming that this segment would likely be highly flexible in the unbound state. Thus the static picture provided by the crystal structure analysis could not unravel how the VWC domain can bind BMPs with high affinity [23]. The determination of the structure of CV2 VWC1 in its unbound conformation using NMR spectroscopy therefore highly complements the data provided by the crystallographic study. The NMR structure first shows that the N-terminal Clip segment as well as the C-terminal subdomain 2 are flexible and partly disordered, whereas subdomain 1 exhibits a small and rigid three-stranded β-sheet core. Secondly, despite the fact that the N-terminal part of the Clip segment is disordered, the NMR structure revealed that the general orientation of the Clip peptide is pre-defined such that the paperclip or hook-like architecture of the Clip and the subdomain 1 is readily formed before binding to BMP ligands. This pre-orientation brings the Clip peptide in close proximity to its final binding site on the BMP ligand thereby very likely reducing the entropy cost that would otherwise be required for fixation of a fully flexible non-oriented Clip. As the key element for localizing and orienting the Clip we could identify the conserved disulfide bridge between the first and the fourth cysteine residue. Its disruption has indeed the same effect on BMP binding as the mutational deletion of these Clip residues. So on this basis do we understand the molecular mechanism of protein recognition for the VWC domain-BMP interaction? So far two structures of VWC modules are known [23,44], of which for one the structure is known for the bound and unbound state. A comparison reveals that the structures differ significantly explicitly in elements required for BMP binding and recognition. Sequence analysis suggests that (at least) two structural classes of VWC domains might exist leading to these two different architectures and hence their BMP binding mechanism might differ. Thus, the VWC1 domain of Crossveinless 2 seems structurally rather an exception compared to the other von Willebrand type C domains known to interact with BMPs. Therefore its mode of BMP recognition and binding might not be transferable to other VWC domains of different BMP modulator proteins. This notion is also supported by the fact, that the Crossveinless 2 VWC1 domain exhibits a uniquely high BMP affinity, whereas the other VWC domains studied by interaction analysis have BMP affinities being 10 to 100-fold lower [24]. In these cases the high affinities for BMPs required for the BMP modulator proteins to efficiently antagonize BMP activity is usually achieved by cooperative binding of several VWC modules of a modulator protein containing multiple VWC domains. Thus the N-terminal von Willebrand type C domain of Crossveinless 2 might present an example where the cooperative binding of several VWC domains has been evolutionarily condensed into a single cooperatively acting VWC domain to allow for efficient inhibition of BMP activity by a single VWC domain.
